# Moving Away from Nasty Encounters Enhances Cooperation in Ecological Prisoner's Dilemma Game

**DOI:** 10.1371/journal.pone.0027669

**Published:** 2011-11-23

**Authors:** Te Wu, Feng Fu, Long Wang

**Affiliations:** 1 Center for Systems and Control, State Key Laboratory for Turbulence and Complex Systems, College of Engineering, Peking University, Beijing, China; 2 Program for Evolutionary Dynamics, Harvard University, Cambridge, Massachusetts, United States of America; University of Maribor, Slovenia

## Abstract

We study the role of migration in the evolution of cooperation. Individuals spatially located on a square lattice play the prisoner's dilemma game. Dissatisfied players, who have been exploited by defectors, tend to terminate interaction with selfish partners by leaving the current habitats, and explore unknown physical niches available surrounding them. The time scale ratio of game interaction to natural selection governs how many game rounds occur before individuals experience strategy updating. Under local migration and strong selection, simulation results demonstrate that cooperation can be stabilized for a wide range of model parameters, and the slower the natural selection, the more favorable for the emergence of cooperation. Besides, how the selection intensity affects cooperators' evolutionary fate is also investigated. We find that increasing it weakens cooperators' viability at different speeds for different time scale ratios. However, cooperation is greatly improved provided that individuals are offered with enough chance to agglomerate, while cooperation can always establish under weak selection but vanishes under very strong selection whenever individuals have less odds to migrate. Whenever the migration range restriction is removed, the parameter area responsible for the emergence of cooperation is, albeit somewhat compressed, still remarkable, validating the effectiveness of collectively migrating in promoting cooperation.

## Introduction

Evolutionary game theory, as a powerful mathematical framework, has been widely employed to model and elucidate the issues surrounding the evolution of cooperation among selfish individuals in disciplines of biology, social science, and economics [1]–[19]. In these fields, the prisoner's dilemma game has become a prominent paradigm to metaphorize the cooperation problem. In a typical prisoner's dilemma, two players simultaneously decide to cooperate or not. A cooperator incurs a cost 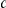 while brings a benefit 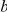 to the other player. A defector (i.e., not to cooperate) bears no cost and generates no benefit. Parameters 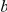 and 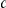 satisfy 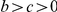. If they have both cooperated, the group's payoff is maximized. But sticking to defection is always better for a given player irrespective of the other's strategy. The disagreement between the best strategy for egoistic individual and that for the group leads to the social dilemma. In addressing this conundrum, many studies have assumed that individuals play the round-robin tournament against all others in the population and accumulate payoffs [20]–[23]. The fitness positively correlated to one's payoff determines its survival (reproducing offspring or replacing others) in the next generation. In this setting, natural selection proceeds at a rate much slower than that interaction happens, since every two individuals play the game one time before competing to survive. Recent experimental studies have shown that this is not always the interaction pattern, especially in biology [24]–[26]. Motivated by these studies, the authors [27] investigated how different time scales between selection and interaction influence individuals' evolutionary fate. In particular, an adjustable quantity, which defines how many number of paired individuals interact before selection occurs, uniquely controls the time ratio of selection to interaction. The results demonstrated that rapid selection can qualitatively alter the properties such as the stability of, the time to arrive at, or the attraction basin of some equilibrium points. In Ref. [28], Woelfing *et al*. also probed the effect of interaction pattern on the ultimate destiny of individuals under different selection strength, and found that the fixation probabilities rather resemble one another for weak selection, whereas strong selection in general results in nonnegligible difference between them.

Along with the well-mixed culture, games on structured populations also have been extensively studied since the pioneering work [29], [30]. In these studies [29]–[37], a standard hypothesis is that each site in the graph carries one individual and, edges determine who plays with whom. Individuals each obtain an aggregate payoff by playing games with all his directly connected neighbors. After that, they simultaneously experience strategy updating and are more likely to learn the strategies of their neighbors with better performance. This is the so-called synchronous updating. Differently, asynchronous strategy updating means that two connected individuals are chosen, and each accumulates his payoff by interacting with all his neighbors. The payoff determines the viability. This pattern is widely employed in investigating the coevolution of strategies and social ties [38]–[46]. Individuals are able to adjust their interacting partners based on the game outcome. Specifically, individuals are disposed to kick off their defector neighbors if opportunities arise, and attempt to relink to altruistic cooperators. The adjustment of neighborhood in turn affects the possible payoff for the future interaction. The coevolution as a feedback mechanism can greatly promote cooperation [38]–[40], [42], [44], [45].

Migration, as a way of realizing the coevolution, has received mounting attention recently [47]–[49]. Instead of actively severing a defecting neighbor, the focal individual terminates interacting with this neighbor by deserting the current location, and moves to an available niche which promises many cooperator neighbors, just as Helbing *et al*. have pointed out that “individuals prefer better neighborhoods, e.g., a nicer urban quarter or a better work environment” [47]. Under the guidance of this idea, the authors constructed a model of success-driven migration. Before imitating the best performing neighbor, the chosen individual is allowed to explore all the adjacent empty sites within an assigned distance, and migrate to the one which potentially brings him the highest payoff. The cost of testing these empty sites is neglected since it is considered very small (named “fictitious play” in reference [47]). It has been demonstrated that cooperation can break out under noisy conditions. We nonetheless think that endowing individuals with the capability to prefigure all these empty sites in their certain neighborhoods constitutes a key element in enhancing cooperation, and seemingly a little strong requirement. We here would like to relax this assumption and investigate whether migration based on only the outcome of previous interaction can stabilize cooperation. In line with the Ref. [47], the pairwise interaction is also modeled in terms of the prisoner's dilemma game. Instead of letting individuals interact with all their neighbors, we in each round randomly pick up a pair of connected individuals and let them play the two-person prisoner's dilemma game and get respective payoffs. According to the obtained payoffs, individuals can infer what strategies the opponents have adopted, and then decide whether or not to migrate. There are three possibilities with respect to the two individuals' strategy combination. If both are cooperators, they stay where they reside. If one is cooperator and the other defector, the cooperator would be privileged to leave its current habitat and move to another permitted empty site, while the defector stays motionless. If the two individuals are defectors, they migrate with equal likelihood. In a generation, a certain fraction of individuals get the chance to interact and migrate. At the end of each generation, all individuals update their strategies by learning more successful neighbors with larger probabilities. We find that in this simple mechanism devoid of large information-processing requirements, the collective migration can uphold cooperation substantially.

### A minimal ecological-evolutionary model

The artificial world is represented by a two-dimensional square lattice with periodic boundary conditions. Of all these 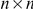 sites, half of them are occupied by individuals, and the rest are left empty. 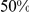 of the individuals are cooperators and the remaining 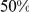 defectors. They are randomly distributed at the beginning. In each round two connected individuals are selected to play the prisoner's dilemma game. Based on the game result, individuals can speculate what strategies their opponents have adopted, and then decide whether or not to abandon their current locations and explore new ones. This exemplifies many biological instances [48], [50]. When a resource niche is overly exploited, animals feeding on it would try to seek some favorable ones rather than stay in this adverse living environment. Also there is no lack of evidence in human society [47], [49]. People are often willing to leave their current interacting partners of being selfish, and seek better ones to improve their welfare. Defectors always provoke dislikes and are most likely to be disassociated. Thus when a cooperator encounters a defector, the former can unconditionally migrate if allowed. When two defectors are confronted, one of them is randomly chosen to move.

As for how to migrate, a dissatisfied individual is allowed to move to one empty site which is less than or equal to 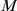 steps from the individual. If in the migration neighborhood of size 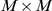 (i.e., a rhombus centered on the individual), there are more than one empty site, then one of them is randomly picked up to accommodate the migrator. If no empty sites exist, the exploited individual has no other choice but to remain in where he is placed. Specially, 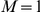 means that each dissatisfied individual can move to one of the empty sites if they exist in his von Neumann neighborhood [51]. The restraint on migration range obviously incorporates the ability of individuals' cognizance. We can actually anneal this restraint by enlarging the range. If dissatisfied individuals are allowed to move to one of all empty sites in the whole space, the migration can always be realized, which we define as the global migration. In this situation, the limitations on individuals' migration ability and cognizance capability are completely neglected. In terms of solitary individuals who have no neighbors, they would hop to one of their four adjacent empty sites when selected. This happens with a small constant probability (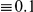), since it takes time for defectors to realize that they have been completely secluded.

Before individuals update their strategies, up to 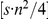 rounds of interaction take place where the Gauss function 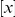 denotes the maximal positive integer less than or equal to 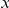. In this situation each individual averagely has a chance of 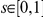 to play the game for one time. Every individual stores an accumulated payoff. Individuals are updated synchronously. Specifically, an individual 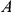 randomly chooses one of his neighbors (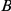), if they exist, and copies 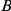
's strategy with the probability given by the Fermi function 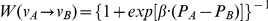, where 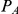 and 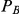 denote the payoffs, and 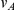 and 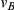 strategies of individuals 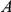 and 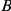, respectively. The parameter 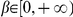 regulates the selection strength. The larger the 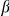, the more important role the payoff difference plays in deciding the selection gradient (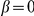 means neutral drift, while 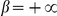 indicates deterministic learning). Solitary individuals keep their strategies unchanged. At the beginning of each generation, all individuals' payoffs are set to be zero. The two-step cycle is repeated before the population arrives at an equilibrium state. It should be noted that in the evolutionary process, the population size remains constant.

Our philosophy is similar to Dirk *et al*'s [47], but differs in a decisive manner. The consistency rests on the migration itself. Differently, the incentive motivating individuals to move in Ref. [47] is based on expectation of the quality of all neighborhoods within a certain distance. Individuals can have access to the quality at a negligible cost. In our model, individuals move just because they are dissatisfied with their partners, with whom they have just interacted. Individuals are totally ignorant of the action of those who they have not interacted or interacted with before the previous round. Apparently as far as time ordering is concerned, future and past are the two sides of the same coin (i.e., now). Moreover, we introduce the parameter 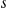 into our model to govern the time scale between interaction and selection. Natural selection acts on all individuals bias-free, yet the number of games that individuals participate in may vary over time because of the different viscosity resulting from the migration. Averagely speaking, each individual gets involved in 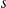 interactions. Therefore, we naturally marry the time scale ratio of interaction to selection with the migration mechanism. This is quite different from most previous studies [39], [40], [42], in which at most one individual has the chance to change strategy but all other ones do nothing concurrently. Central issues concerning our interest include effects of varying time scale, different migration range, and the selection intensity on the evolution of cooperation.

## Results

### Effect of migration on the evolution of cooperation

**Figure 1:**
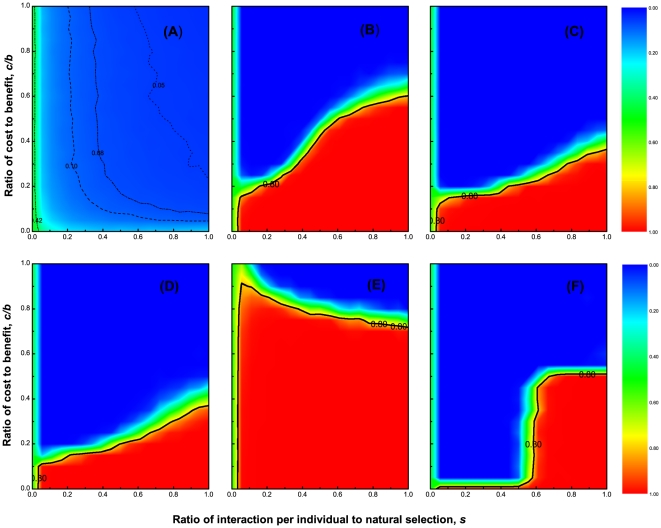
Density of cooperators at equilibrium state as a function of the combination of 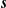 and 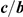. These panels are related to different migration patterns: 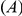 no migration, 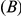 local migration with 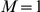, 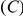 local migration with 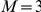, and 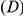 global migration, under strong selection 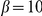. Those ones are related to different selection intensities: 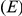 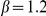, 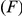 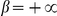, under local migration pattern of 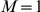. Whenever individuals are prohibited to move, the empty sites play the role of demarcating cooperators and defectors, leading to that a few small but not many cooperator clusters survive the evolutionary race. This does not alter the fact that cooperators are in a disadvantageous place in contending with defectors. Integrating the migration into the system qualitatively changes the evolutionary outcome. Under the 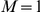 migration pattern, a large area sees red (full cooperative), reversing cooperators' fate. Under global migration pattern, the area surviving cooperators is somewhat compressed, but is still much larger than the situation of no migration. Comparing panels 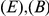 and 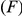 shows that increase in 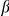 proves to be adverse for the evolution of cooperation. The red area tends to drop down as 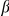 increases. Of interest is that for large 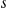, cooperation can always be stabilized at a markedly large tract, even for 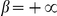. For small 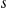 the red area nonetheless plummets much more rapidly for increasing 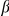. In terms of strategy revision, 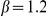 adds the stochasticity, offering much longer time span for cooperators to agglomerate. This constitutes a key reason why cooperators absolutely outperform defectors for relatively weak selection (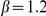).

We first illustrate the results related to the evolutionary dynamics of the population in the absence of migration mechanism, which can be regarded as a reference (see panel 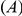 in Fig. 1). The dynamics can be generally divided into three regimes based on the fraction of cooperating individuals in the steady state. For 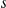 approaches zero, a few but not many individuals have chance to participate in the game and accumulate inappreciable payoffs. Most other individuals accrue zero payoff. Neutral drift predominates in individuals' strategy updating. As a result, cooperators and defectors each account for approximately 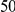 percentage of the population, independent of the change in the ratio of cooperation cost 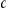 to cooperation benefit 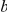 (see the long narrow slot of light blue in color along the vertical axis). As 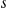 bulges, individuals' payoffs coming from playing game navigate the direction of natural selection. At this time, for little value of 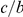, equivalent to relatively weak advantage of defection over cooperation, cooperators can always, albeit inferior in numbers, survive. The presence of empty sites plays the role of completely separating cooperators from defectors, leading to the coexistence state, which is not a dynamical but a frozen equilibrium, as there is not strategy switching or migration any more in this state. Moreover, giving individuals more chance to play the game lowers the cooperation level.

Individuals, especially advanced primates, possess instinct to escape from unfavorable habitats. We here equip individuals with ability of migrating. Three typical scenarios in terms of the migration range are investigated. 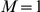 indicates dissatisfied individuals can just move to the directly connected empty sites. Under this limitation, the migration cannot always be accomplished. The most adverse situation is that a cooperator is surrounded by 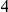 defectors. The range 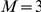 indicates that one dissatisfied individual can move away if there are empty ones among all the 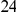 sites, which is an almost sure event. Both 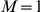 and 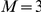 are spatially restricted patterns. Global migration is the spatially unrestricted pattern. The average level of cooperation at equilibrium is statistically recorded and presented as panels 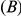, 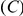 and 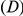 in Figure 1 corresponding to the three scenarios respectively. Generally speaking, there exist two threshold values, 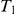 and 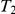, which depend on the typical migration pattern. For 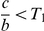, cooperators successfully spread and eventually prevail in the entire population. In diametrical contrast, for 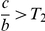, the population resides at a state teeming with all defectors. Intriguingly, for medium 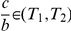, depending on 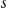, the population sees three strikingly different regimes: for small 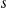, defectors homogenize the entire population, while cooperators and defectors coexist for a narrow interval of moderate 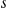, and further increment in 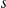 puts cooperators in the advantageous place and thus makes them thrive. Under the 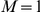 migration pattern, 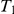 and 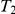 are maximized, and the red area, in which the cooperation level is above 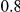, covers saliently larger fraction of the plot than the other two patterns. Therefore, the restraint on the migration range as 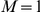 most favors the evolution of cooperation. It is worth emphasizing that the population exhibits no essentially different ecological-evolutionary dynamics between 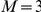 and the global migration.

**Figure 2:**
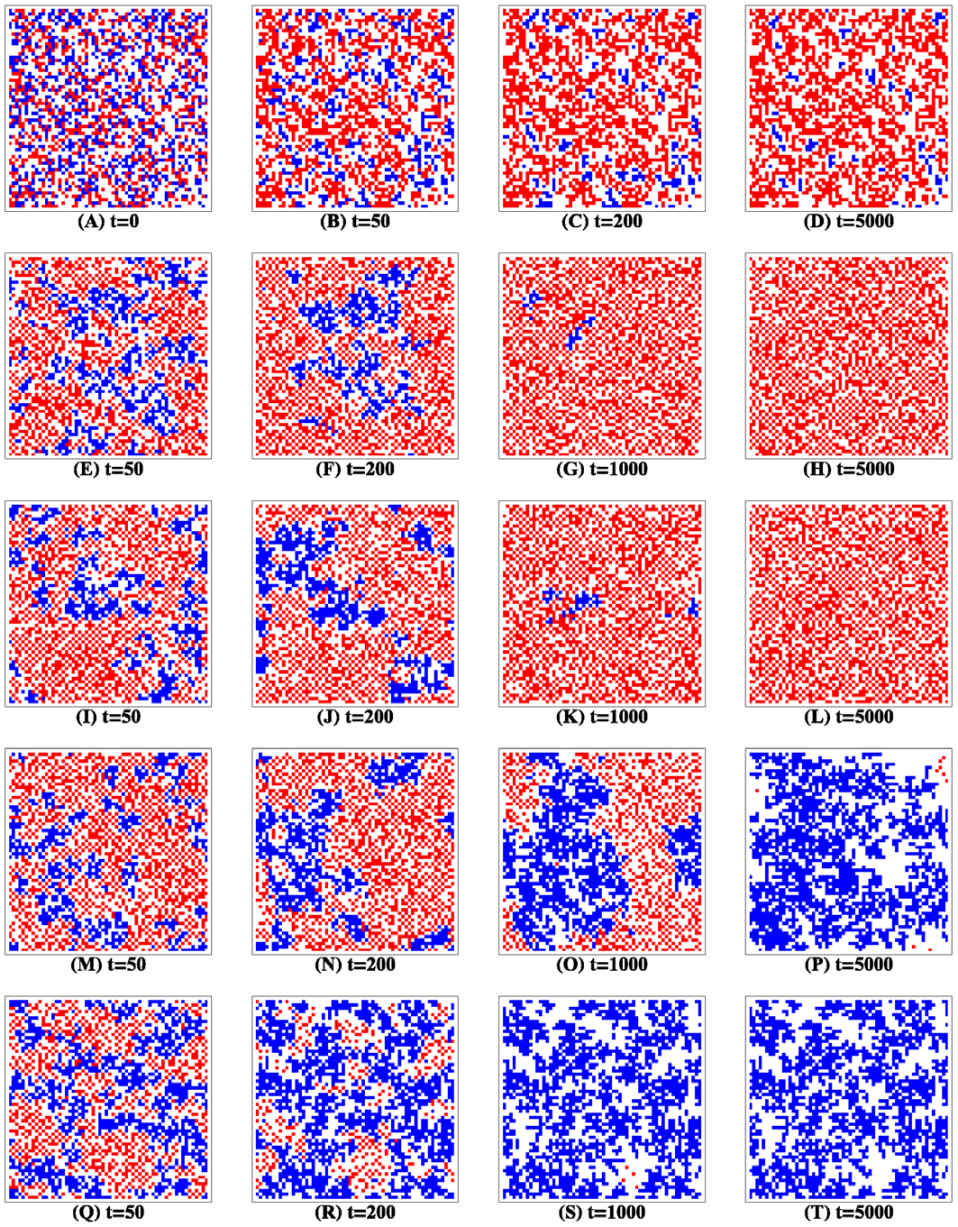
Distribution of cooperators and defectors in a typical evolutionary process for the prisoner's dilemma. All simulations were conducted on a square lattice of size 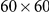 with 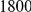 individuals, half of which are randomly initialized as cooperators (see plot 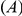). Red, blue and white cell denotes cooperator, defector and empty site respectively. Just isolated individuals are allowed to migrate to the neighboring empty sites (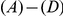), but dissatisfied individuals are also allowed to move to one-step away empty sites if they do exist (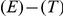). Each row is for the same process at different time step. The time scale ratio 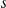 varies with row. In the absence of migration, only those cooperators who are totally secluded from defectors can survive (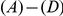). Cooperators are doomed under rapid natural selection (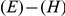: 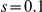). For moderate 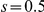, either defectors win the evolutionary race (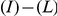), or cooperators get established (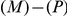). Averagely speaking, they coexist. Cooperators always pervade the whole population under slow natural selection (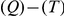: 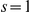). Other parameters: 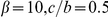.

To understand these observations, we now probe into the microscopic evolutionary process. Snapshots of the distribution of cooperators and defectors are presented in Figure 2. Panels 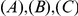 and 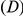 show the change in the distribution in the absence of migration as the evolution proceeds. The number of cooperators drastically decreases at the starting phase. Occasionally, several small clusters of cooperators form. Only completely isolated defectors are likely to invade these stable clusters. Actually such defectors are quite sparse. If they arrive at the periphery of defector clusters, they cease moving. If they move to the periphery of cooperator clusters, they either invade the clusters successfully or are assimilated by the cooperators. It is impossible for them to coexist in the long run. Thus several cooperator islands survive permanently. Larger 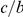 accelerates defectors' replacing cooperators, who are unable to cluster quickly enough against the invasion. Defectors permeate into the whole population.

Incorporating the migration mechanism into the population, the role of empty sites completely preventing cooperators from being invaded by defectors is removed. How quickly cooperators cluster and how large these clusters are determine the ultimate fate of cooperators. Whenever each individual on average gets a fat chance (i.e., 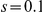) to involve the game, natural selection takes place more rapidly than interaction. In each generation, repeated interactions among the same pairwise neighbors are negligible, therefore cooperation-enhancing force due to direct reciprocity is hardly existential. In the incipient phase of evolution, a few small clusters of cooperators indeed come into being, while most of sparsely distributed cooperators are quickly defeated and demoted to defectors. As the number of defectors increases, many defector-defector links produce, reducing the probability that cooperators feed cooperators. As a consequence, the network reciprocity based on the clustering of cooperators is greatly inhibited. Furthermore, expulsion between defectors makes them repulse each other. Defectors incessantly move for the sake of seeking cooperators to exploit. Defectors' migration often destroys the stability of these small cooperative communities, which inevitably shrink over time and eventually die out.

As 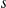 appreciates to moderate values, the evolutionary process exhibits remarkably different properties. At the outset, a number of cooperator clusters of varying size build up, of which the small ones are unable to defend the aggression of contiguous defectors and disappear as above analyzed. As for the slightly large ones, whether they can survive depends on where defectors invade them. From time to time, defectors are likely to permeate the cooperator clusters, which are coerced to split into many ones of varying size. If they cannot gather quickly enough to defend defectors' invasion, these small cooperator patches vanish, and defectors become the unique survivors. However, if it happens that some of these clusters have expanded into some larger ones before defectors are able to disintegrate them, the strong assortment of cooperators makes them generally win the contest with the vagrant defectors, and thereafter assimilate them. Consequently, these closely huddled cooperators spread out and further enlarge their territories, and progressively pervade into the whole space. Averagely speaking, cooperators and defectors coexist.

Further aggrandizing 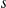 expedites the rate at which the interaction proceeds and dissatisfied individuals migrate. More cooperator clusters emerge. Almost all defectors are rendered to be isolated. Given that each round a pair of individuals are picked to interact, the isolation of defectors implicitly indicates that more interactions befall upon cooperators, reinforcing the positive effect of cooperator correlation and raising the likelihood of cooperators repeatedly encountering. Once defectors travel to the margin of the clusters, they are immediately homogenized as cooperators, leading to concomitant expansion of cooperators' territories. Ironically, for defectors, the expulsion between interspecies accelerates the mortality of themselves.

Insofar, we have found that just introducing empty sites into the population slightly promotes cooperation, which is totally due to the quarantine of a few cooperator clusters from defectors by these empty sites. Whenever migration is incorporated, the insulating effect is ruled out. Cooperation can be substantially improved for a wide range of model parameters. For small 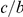, cooperation constitutes the unique evolutionarily stable strategy (ESS) [3] conditioning that 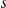 is above a very small threshold value. For large 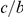, defectors prevail. For the value of 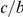 between these two extremes, scenarios of defector dominance, coexistence, or cooperator dominance possibly arise as 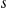 varies. Although coexistence emerges both for small 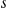 near to zero and moderate 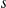, the rationale behind this phenomenon is quite different. In the former case, inconspicuous number of interactions occur, thus neutral drift steers the direction of the strategy selection. In the latter case, natural selection decides their evolutionary fate, and the migration mechanism makes both cooperators and defectors have chance to dominate. From perspective of statistics, they coexist. For 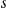 lying in between, relevant area in the plot displays deep blue (i.e., zero cooperation level), confirming that migration does destroy the role of empty sites serving as “insulators”, which can sustain a low level of cooperation. Whenever each individual averagely has one chance to play the game (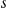 approaching one) and migrate, the strong assortment between cooperators and the expulsion towards defectors make cooperators invariably win the evolutionary race.

The relaxation on the migration range 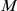 makes the implementation of migrating always achievable. The limitation-free migration indubitably can expedite cooperators' agglomeration, favoring the achievement of network reciprocity between cooperators. But we should not forget that defectors possess the same property of migrating. Defectors' global migration exacerbates their aggression into cooperator patches, impeding cooperators' gathering. Especially whenever defectors move to the internal empty sites of cooperator clusters, who are probably forced to disintegrate into several small ones. These waning cooperator clusters become dampened in resisting invasion of defectors. In the tug of war between the two polar effects, the negative one greatly counteracts the positive one. This accounts for the striking diminution of the area promoting cooperation (see panels 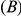 and 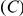 in Fig. 1).

### Role of selection intensity in the evolution of cooperation

Getting motivation from the experimental findings [52], we have also explored how varying selection intensity affects the evolution of cooperation under the 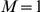 local migration. Results show that the less the 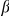, the larger area promoting cooperation. Under the most realistic situation (i.e., 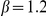), cooperation is substantially promoted (see panel 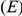 in Fig. 1). And there exists an optimal 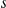, which ensures steady state being full cooperative even 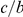 approaches 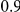. This observation can be mainly ascribed to two fronts. On the one hand, the decrease in 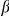 adds the stochasticity of strategy learning, offering longer time available for individuals, especially for cooperators to forage for identical species, before the population gets stabilized. The formation of cooperator clusters enhances cooperators' capacity to prevent themselves from being invaded. On the other hand, there is enough chance to realize the expulsion between the defectors. The dilution of defector communities makes cooperators have more chance to undergo gameplay, as well as reduces the likelihood of defectors serving as model individuals. Actually, we have found that for weak selection 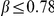, cooperators always win the evolutionary race under the 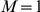 migration pattern.

In the limit of the strong selection 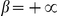, the less fit one deterministically adopts the fitter' strategy. The evolutionarily stable outcomes see drastic change. For small 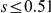, some small cooperator clusters indeed form at the beginning. There is not enough time space for defectors to exclude each other. Interactions befalling upon defectors tends to lower chance that cooperators mutually bring benefit. Defectors' interaction accrues each protagonist a benefit of 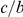 instead of zero. Because of just storing the information concerning previous-round interacted opponents, migration is not always favorable for cooperators, who have to pay the cost of being exploited for each migratory move. Even worse, some cooperators are likely to move forward and backward between two nasty neighborhoods when defectors are prevalent. It is demanding for cooperation to emerge. Taken together, only when both social dilemma is relatively weak (small 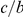) and individuals experience selection at a slow rate (large 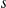), can cooperation evolve. Otherwise, cooperators are always doomed (see panel 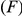 in Fig. 1).

Interestingly, as 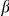 increases from 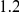 to 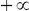, the cascade of red area contracts, meaning that the cooperation-enhancing force resulting from migration is compromised. The increase in 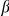 demotes the viability of cooperators at different speed for different 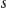. The less 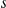, the harder it is for cooperation to establish with 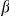
's increasing. Once 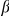 exceeds a threshold value, the positive effect of migration for the evolution of cooperation is completely neutralized. In contrast, whenever each individual is averagely able to play the game one time, cooperation can be maintained for a large range of 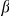. Even under the most adverse situation (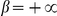), cooperation can still emerge for 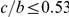 (see panel 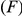 in Fig. 1).

## Discussion

We would like to compare our model with some intimately related ones. In Ref. [50], it is demonstrated that local migration weakens the competition, thus favoring the survival of those who prudently use the common resource, whereas the unrestricted migration allows one type to exclude the others. Generally speaking, cooperation in our model can be seen as the prudent use, while defection the over-exploitation of a limited common resource. Our results have illustrated that local migration promotes the evolution of cooperative behavior, consistent with the findings [50]. Furthermore, we have found qualitatively similar phenomena that defectors fare better in the global migration (equivalent to unrestricted migration in [50]) whereas cooperators turn out to be the ultimate winner under local interaction pattern (both competition and migration) for moderate 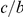. Differently, for relatively weak advantage of defectors over cooperators, migration, either local or global, is always able to establish cooperation under strong selection, suggesting that migration benefits cooperators more.

In a broad sense, the migration can be seen as the coevolution of strategy and social ties. Comparing with previous studies pertaining to coevolution [38], [40], [42], [44], there are three striking differences. Apart from individuals in the population, we also introduce empty sites, which can stand for available resource niches, shelters, and so forth. At the same time, we just pick up two connected individuals to play the game rather than let them interact with all their directly connected individuals. But up to a certain number of pairs are selected in each generation. Thus the time scale ratio of game interaction to strategy updating is naturally integrated. Payoffs are also accumulated, which differs from the literature [38], [40], [42], [44] in which just a pair of individuals are involved but their neighbors would not accumulate the payoff, and in this process all other individuals do nothing. Furthermore, instead of kicking off defecting neighbors, in our model dissatisfied individuals choose to move away. The physical network are fixed but the specific locations of individuals are time-changing. This reflects such a scenario where environment is unlikely to change, but individuals are able to decide where to live [49]. Although individuals are randomly distributed on the network, neighborhoods of varying quality differ in attracting individuals' settlement, leading to the diverse population viscosities from patch to patch. Individuals therefore engage in inhomogeneous rounds of game. This heterogeneity is a key factor promoting cooperation. Under strong selection, strategy replacement happens at a rapid rate. Only very strong assortment of cooperators can make them win the competition with defectors. To arrive at this assortment, large 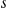 is indispensable. Cooperation still builds up for small 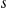, but that just holds for very low 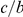. Thirdly, it is the collective migration that promotes cooperation. Unlike the assumption that individuals are able to speculate in Ref. [47], the information that individuals can acquire is quite limited in the present work. Even they successfully migrate, there is no guarantee they can neighbor with cooperators. Owing to this uncertainty, the collective coordination of migrating is overridingly crucial to the emergence and persistence of cooperation.

The incentive in our model for individuals to migrate is very simple. They move away if they have been exploited by defectors. Thus the achievement of migrating is costly. The gist of the migration mechanism is to “win stay, lose migrate”, which aims at reducing the exploitation of the selfish partners. We could not absolutely preclude the possibility that some cooperators swing between two nasty neighborhoods. In this perspective, the migration is risky. This to some extent discounts the effectiveness of the goal of cooperators' leaving defectors. However, our results suggest that unknown niche exploration turns out better than awaiting continual exploitation for cooperators. As we have allowed unprejudicedly dissatisfied individuals to leave the current habitats, actually, cooperators run ahead, and defectors chase behind in the process of evolution. The local migration promotes the competitive restraint, leading to that the short-term prosperity of defectors trades off with the long-term persistence. Whenever defectors arrive at the periphery of cooperators, they are either absorbed or displace the peripheral cooperators. If the latter, the clumping of defectors in turn breaks up the stability of the defective communities. Under one-step local migration, these defectors are unlikely to hunt cooperators quickly and efficiently, which leaves cooperators with sufficient odds to agglomerate by the trial-and-error-based migration. The clustering of cooperators is positively correlated to their stability. Cooperators located in these clusters are able to evolve higher levels of competitiveness and capacity in resisting invasion. Combining these considerations together, range-restricted migration promotes the evolution of cooperation in quite a large tract of the model parameters.
